# A Report of Two Cases: Unlearning Lactic Acidosis

**DOI:** 10.5811/cpcem.2021.2.51634

**Published:** 2021-04-08

**Authors:** Sanjay Mohan, David S. Goldfarb, Robert S. Hoffman

**Affiliations:** * NYU Grossman School of Medicine, Ronald O. Perelman Department of Emergency Medicine, New York, New York; † NYU Grossman School of Medicine, Division of Nephrology, New York, New York

**Keywords:** lactate, anion gap, acidosis, alkalosis

## Abstract

**Introduction:**

The term “lactic acidosis” reinforces the misconception that lactate contributes to acidemia. Although it is common to discover an anion gap acidosis with a concomitant elevated lactate concentration, the two are not mutually dependent.

**Case Report:**

Here we describe two patients exhibiting high lactate concentrations in the setting of metabolic alkalemia.

**Conclusion:**

Lactate is not necessarily the direct cause of acid-base disturbances, and there is no fixed relationship between lactate and the anion gap or between lactate and pH. The term “metabolic acidosis with hyperlactatemia” is more specific than “lactic acidosis” and thus more appropriate.

## INTRODUCTION

Demystifying acid-base disorders is a staple of medical education and clinical practice.^1^,^2^ Year after year, medical students, house staff, and senior clinicians alike tackle the intricacies of anion gaps and delta gaps, and apply Winters’ formula, all in an effort to elucidate the metabolic and respiratory status of their patients.^3^ More specifically, when confronted with a metabolic acidosis of unclear etiology, mnemonics such as “MUDPILES” or “KULTS” are often used to develop and refine a differential diagnosis.

Any discussion of acid-base disturbance inevitably leads to discourse regarding the contribution of lactate. Serial monitoring of lactate has become a cornerstone in the management of the critically ill. Lactate is commonly used to delineate the severity of sepsis, interpreted as a prognostic marker, and regarded as a surrogate for tissue perfusion.^4^,^5^ Under most circumstances, an elevated lactate concentration implies impaired tissue perfusion. However, high lactate concentrations may represent toxin-induced impairment of cellular metabolism without overt evidence of systemic hypoperfusion; examples include metformin, cyanide, and carbon monoxide to name a few.^6^

Given the ubiquity of lactate and metabolic acidosis in clinical practice, it is common parlance to use the term “lactic acidosis” when both conditions are present. However, we believe that this term reinforces the misconception that lactate itself contributes to acidemia. Instead, we prefer “metabolic acidosis with hyperlactatemia” to recognize two coexistent events that are not necessarily directly related. To illustrate this concept, we describe two patients exhibiting high lactate concentrations in the context of profound metabolic alkalosis. We will then discuss the relationship between lactate and acid-base physiology.

## CASE REPORTS

### Case 1

A 48-year-old man with metastatic hepatocellular carcinoma presented to the emergency department (ED) with three days of nausea and vomiting. He was discharged from an outside facility one month prior and was placed on home hospice care. For the three days preceding admission, he had intermittent fevers and nausea, and persistent episodes of profuse non-bloody, non-bilious emesis. During this time, the patient was unable to tolerate liquids or solids by mouth.

On physical examination, his vital signs included a blood pressure of 87/49 millimeters mercury (mm Hg), a pulse of 128 beats per minute, a respiratory rate of 18 breaths per minute, a temperature of 103.2^o^F, an oxygen saturation of 99% (room air), and a point-of-care glucose of 88 milligrams per deciliter (mg/dL) (Reference Range: 70 – 100 mg/dl). He was ill appearing, cachectic, jaundiced, and oriented to name only. Mucous membranes were dry. Heart and lung examinations were unremarkable aside from tachycardia. His abdomen was soft and nontender, and melena was noted on rectal examination. The initial laboratory data is shown in the table.

Other pertinent findings included leukocytosis, anemia, hyperbilirubinemia of 15.9 mg/dL (reference range: 0.2 – 1.2 mg/dL and elevations of aspartate and alanine amino)transferases (228 and 177 IU/L, respectively; reference ranges: 5 – 34 IU/L and 0 – 37 IU/L respectively).

Blood and urine cultures were obtained and the patient was started on broad spectrum antibiotics. Volume resuscitation consisted of two liters of 0.9% sodium chloride. Approximately two hours later, a repeat venous blood gas demonstrated a pH of 7.51, a partial pressure of carbon dioxide (pCO_2_) of 42 mm Hg, and a lactate of 10.6 millimoles per liter (mmol/L). In discussion with the family, the patient was given only comfort care and no subsequent laboratory values were drawn. Antibiotics were discontinued two days after admission, and the patient expired one week after initial presentation. Blood cultures grew *Enterococcus faecalis*.

### Case 2

A 39-year-old woman with chronic back pain was brought to the ED for altered mental status. According to family, she became depressed over the prior few months as a result of unemployment and the pandemic. Over the preceding two days, she had stopped eating and began to have episodes of non-bilious, non-bloody emesis. On the morning of presentation, she was found unresponsive at home and emergency medical services were called.

On physical examination, her vital signs included a blood pressure of 107/80 mm Hg, a pulse of 100 beats per minute, a respiratory rate of 16 breaths per minute, a temperature of 98.3^o^F, an oxygen saturation of 100% (bag valve mask on 10 L of oxygen), and a point-of care glucose of 93 mg/dL. On general inspection, the patient appeared to be an incoherent, mumbling woman with poor hygiene and dried vomitus around her mouth. She was tachycardic, with normal heart sounds and clear lungs. Abdominal examination was notable for diffuse tenderness. She was subsequently intubated for airway protection. The initial laboratory data is shown in the table.

**CPC-EM Capsule**

What do we already know about this clinical entity?

*Serial monitoring of lactate is a cornerstone in the management of the critically ill patient and is often associated with a concomitant acidemia*.

What makes this presentation of disease reportable?

*We report the infrequent occurrence of an elevated lactate in the context of a profound metabolic alkalemia*.

What is the major learning point?

*The presence of lactate does not inherently indicate a coexisting acidemia. In fact, there is no fixed relationship between lactate and the anion gap*.

How might this improve emergency medicine practice?

*“Lactic acidosis” implies that lactate contributes to an acidemia. Instead, “metabolic acidosis with hyperlactatemia” is more appropriate in education and practice*.

Other than leukocytosis the rest of her laboratory evaluation was not contributory. Blood and urine cultures were drawn. The electrocardiogram was significant for a prolonged absolute QT interval of 557 milliseconds (reference range in women: < 460 milliseconds). Computed tomography of the head, chest, abdomen, and pelvis was negative for acute pathology. In the ED, the patient received broad spectrum antibiotics, thiamine, two liters of 0.9% sodium chloride, and a total of 80 milliequivalents of potassium supplementation.

Thereafter, she was admitted to the medical intensive care unit (MICU). While in the MICU, blood cultures grew *Staphylococcus aureus* and urine cultures grew *Proteus mirabilis*. The patient was continued on antibiotics, fluids, and potassium supplementation. She was extubated on hospital day two and transferred to the medical floor. Prior to leaving against medical advice on day four, her venous blood gas demonstrated a pH of 7.50, a pCO_2_ of 38 mm Hg, and a lactate of 1.0 mmol/L.

## DISCUSSION

To understand lactate and the associated changes in acid-base status, a brief discussion of both aerobic and anaerobic metabolism is warranted. Glycolysis (summarized in Figure, Equation A) converts one glucose molecule into two molecules of pyruvate. Glycolysis is anaerobic and occurs in the cytosol without the need for mitochondria.

In the presence of oxygen, pyruvate is transported into mitochondria and converted to acetyl coenzyme-A by the pyruvate dehydrogenase complex. Acetyl coenzyme-A then enters the citric acid cycle to drive adenosine triphosphate (ATP) synthesis. However, under anaerobic circumstances, in which oxygen cannot act as the final electron acceptor, pyruvate is converted to lactate in order to regenerate nicotinamide adenine dinucleotide via lactate dehydrogenase (Figure, Equation B). It is noteworthy that the conversion of pyruvate to lactate consumes a proton. Moreover, pyruvate and lactate are close to 100% ionized at a physiologic pH. Thus, the production of lactate in and of itself cannot account for the acidemia that often coexists with an elevated lactate concentration. As is summarized in figure, equation C, following glycolysis there is a net gain of two molecules of ATP without any change in hydrogen ion concentration.

Rather, it is the utilization of ATP under anaerobic conditions that generates a change in pH (Figure, Equation D). Despite this fact, there is a tendency to sum the reactions for anaerobic glycolysis (Figure, Equation C) and ATP hydrolysis (Figure, Equation D) resulting in Figure, Equation E, thus implying that lactate generation is inherently associated with an acidosis. However, it is imperative to recognize that these two processes are not intrinsically related.

When evaluating the differential of an elevated anion gap, classic teaching often stresses that organic acids, particularly lactic acid, should be suspected.^7^ However, the anion gap has a low sensitivity as a screening test for hyperlactatemia; in fact, elevated lactate concentrations should also be included in the differential of a non-anion gap acidosis.^8^ To account for hyperlactatemia in the presence of a normal anion gap, it is necessary to evaluate for hyperchloremia (often due to electrolyte-free water deficits or resuscitation with high chloride fluids), hypoalbuminemia, or the presence of mixed acid-base disturbance. While it is common to discover an anion gap acidosis and acidemia with a concomitant elevated lactate concentration, these two conditions are not mutually dependent.

In fact, alkalemia stimulates lactic acid production.^9^ Several in-vitro studies demonstrated that pH has a profound influence over certain rate-limiting enzymes of glycolysis.^10^ Phosphofructokinase (PFK), one such glycolytic enzyme, is particularly sensitive to pH. Inhibition of PFK by acidosis leads to reduced serum lactate, whereas alkalotic conditions potentiate a rise in lactate.^7^ This mechanism is felt to help normalize pH under anaerobic conditions, allowing for optimal intracellular enzymatic functioning, especially during times of metabolic stress.^11^ In the context of an elevated pH, most elevations in serum lactate concentrations are modest as there is often an equally large increase in hepatic lactate consumption.^12^ When marked elevation of lactate is noted, sepsis, shock, or tissue hypoperfusion are often simultaneously identified.^13^

However, a “true” lactic acidosis is possible in which the presence of an elevated lactate and metabolic acidemia are inherently linked. This is best illustrated by the metabolism of propylene glycol, a pharmaceutical diluent and an antifreeze. Following successive oxidation by alcohol and aldehyde dehydrogenases, propylene glycol is ultimately metabolized to lactic acid.^14^,^15^

## CONCLUSION

Both of the described cases demonstrate profoundly elevated lactate concentrations in the setting of alkalemia. By highlighting these cases and describing the biochemical nuances of energy metabolism, our aim is to disentangle hyperlactatemia and metabolic acidosis. While both conditions often coexist, the presence of one does not inherently indicate that the other is present. The generation of lactate is not directly associated with a change in pH. Rather, it is the hydrolysis and consumption of ATP that creates an acidosis. Moreover, while lactate is considered in the differential diagnosis of an anion gap acidosis, there is no fixed relationship between lactate and the anion gap. Confounding factors such as hyperchloremia, hypoalbuminemia, or mixed acid-base disturbances, should be evaluated as they may lead to hyperlactatemia with a non-anion gap acidosis.

It is our hope that the term “lactic acidosis” be avoided as it implies that lactate itself contributes to an acidemia. Instead, “metabolic acidosis with hyperlactatemia” appears to be more specific and thus more appropriate, both for medical education and clinical practice.

## Figures and Tables

**Figure:**
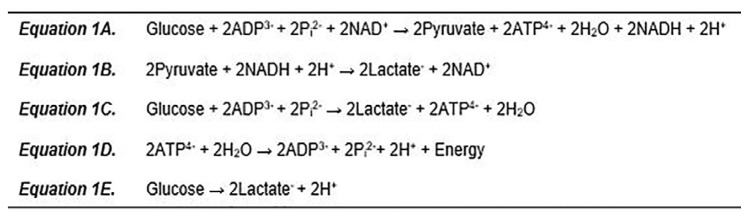
Anaerobic metabolism. *A*. Glycolysis; *B*. Reduction of pyruvate; *C*. Net glycolytic reaction under anaerobic conditions; *D*. ATP utilization under anaerobic conditions; *E*. Summation of anaerobic glycolysis and ATP utilization. *ADP*, adenosine diphosphate; *P*_i_, phosphate; *NAD*, nicotinamide adenine dinucleotide; *ATP*, adenosine triphosphate; *H_2_O*, water; *NADH*, nicotinamide adenine dinucleotide hydrogen; H^+^ hydrogen Ion.

**Table:** Case 1: Initial laboratory studies of patient with hepatocellular carcinoma. Case 2: Initial laboratory studies of patient presenting with altered mental status.

	Na^+^ mmol/L	K^+^ mmol/L	Cl^−^ mmol/L	Bicarbonate mmol/L	BUN mg/dl	Cr mg/dl	Anion Gap	pH	pCO_2_ mmHg	Lactate mmol/L
Case 1	142	4.2	59	38	104	4.7	45	7.58	40	22
Case 2	138	2.4	62	>40	36	1.0	N/A	7.61	60	9.3
Reference Range	136–145	3.5–4.8	98–107	22–29	7–20	0.6–1.1	6–14	7.35–7.45	35–45	0–1.9

*Na*^+^, sodium; *K*^+^, potassium; *Cl*^−^, chloride; *BUN*, blood urea nitrogen; *Cr*, creatinine; *pCO_2_*, partial pressure of carbon dioxide; *mmol/L*, millimoles per liter; *mg/dL*, milligrams per deciliter; *mm Hg*, millimeters mercury.
